# Left atrial deformation analysis in patients with non-ischaemic dilated cardiomyopathy in atrial fibrillation

**DOI:** 10.1093/ehjimp/qyae063

**Published:** 2024-06-25

**Authors:** Eduard Ródenas-Alesina, Jordi Lozano-Torres, Pablo Eduardo Tobías-Castillo, Clara Badia-Molins, Rosa Vila-Olives, Maria Calvo-Barceló, Guillem Casas, Toni Soriano-Colomé, Aleix Olivella San Emeterio, Rubén Fernández-Galera, Ana B Méndez-Fernández, José A Barrabés, Ignacio Ferreira-González, José Rodríguez-Palomares

**Affiliations:** Department of Cardiology, Vall d’Hebron University Hospital, Passeig de la Vall d’Hebron 119-129, 08035 Barcelona, Spain; Department of Medicine, Universitat Autònoma de Barcelona, Passeig de la Vall d‘Hebron 119-129, 08035 Barcelona, Spain; Department of Cardiology, Vall d’Hebron University Hospital, Passeig de la Vall d’Hebron 119-129, 08035 Barcelona, Spain; Department of Medicine, Universitat Autònoma de Barcelona, Passeig de la Vall d‘Hebron 119-129, 08035 Barcelona, Spain; Department of Cardiology, Vall d’Hebron University Hospital, Passeig de la Vall d’Hebron 119-129, 08035 Barcelona, Spain; Department of Medicine, Universitat Autònoma de Barcelona, Passeig de la Vall d‘Hebron 119-129, 08035 Barcelona, Spain; Department of Cardiology, Vall d’Hebron University Hospital, Passeig de la Vall d’Hebron 119-129, 08035 Barcelona, Spain; Department of Medicine, Universitat Autònoma de Barcelona, Passeig de la Vall d‘Hebron 119-129, 08035 Barcelona, Spain; Department of Cardiology, Vall d’Hebron University Hospital, Passeig de la Vall d’Hebron 119-129, 08035 Barcelona, Spain; Department of Medicine, Universitat Autònoma de Barcelona, Passeig de la Vall d‘Hebron 119-129, 08035 Barcelona, Spain; Department of Cardiology, Vall d’Hebron University Hospital, Passeig de la Vall d’Hebron 119-129, 08035 Barcelona, Spain; Department of Medicine, Universitat Autònoma de Barcelona, Passeig de la Vall d‘Hebron 119-129, 08035 Barcelona, Spain; Department of Cardiology, Vall d’Hebron University Hospital, Passeig de la Vall d’Hebron 119-129, 08035 Barcelona, Spain; Department of Medicine, Universitat Autònoma de Barcelona, Passeig de la Vall d‘Hebron 119-129, 08035 Barcelona, Spain; Department of Cardiology, Vall d’Hebron University Hospital, Passeig de la Vall d’Hebron 119-129, 08035 Barcelona, Spain; Department of Medicine, Universitat Autònoma de Barcelona, Passeig de la Vall d‘Hebron 119-129, 08035 Barcelona, Spain; Department of Cardiology, Vall d’Hebron University Hospital, Passeig de la Vall d’Hebron 119-129, 08035 Barcelona, Spain; Department of Medicine, Universitat Autònoma de Barcelona, Passeig de la Vall d‘Hebron 119-129, 08035 Barcelona, Spain; Department of Cardiology, Vall d’Hebron University Hospital, Passeig de la Vall d’Hebron 119-129, 08035 Barcelona, Spain; Department of Medicine, Universitat Autònoma de Barcelona, Passeig de la Vall d‘Hebron 119-129, 08035 Barcelona, Spain; Department of Cardiology, Vall d’Hebron University Hospital, Passeig de la Vall d’Hebron 119-129, 08035 Barcelona, Spain; Department of Medicine, Universitat Autònoma de Barcelona, Passeig de la Vall d‘Hebron 119-129, 08035 Barcelona, Spain; Department of Cardiology, Vall d’Hebron University Hospital, Passeig de la Vall d’Hebron 119-129, 08035 Barcelona, Spain; Department of Medicine, Universitat Autònoma de Barcelona, Passeig de la Vall d‘Hebron 119-129, 08035 Barcelona, Spain; Centro de Investigación Biomédica en Red de Enfermedades Cardiovasculares, Av. Monforte de Lemos, 3-5, 28029 Madrid, Spain; Department of Cardiology, Vall d’Hebron University Hospital, Passeig de la Vall d’Hebron 119-129, 08035 Barcelona, Spain; Department of Medicine, Universitat Autònoma de Barcelona, Passeig de la Vall d‘Hebron 119-129, 08035 Barcelona, Spain; Centro de Investigación Biomédica en Red de Epidemiología y Salud Pública, Av. Monforte de Lemos, 3-5, 28029 Madrid, Spain; Department of Cardiology, Vall d’Hebron University Hospital, Passeig de la Vall d’Hebron 119-129, 08035 Barcelona, Spain; Department of Medicine, Universitat Autònoma de Barcelona, Passeig de la Vall d‘Hebron 119-129, 08035 Barcelona, Spain; Centro de Investigación Biomédica en Red de Enfermedades Cardiovasculares, Av. Monforte de Lemos, 3-5, 28029 Madrid, Spain

**Keywords:** left atrial strain, non-ischaemic dilated cardiomyopathy, atrial fibrillation, left atrial filling index, echocardiography

## Abstract

**Aims:**

Atrial fibrillation (AF) is a common comorbidity in non-ischaemic dilated cardiomyopathy (NIDCM) affecting conventional measures of left atrial (LA) function. We aimed to determine whether LA function analysis could identify patients at higher risk of major cardiovascular events (MACEs).

**Methods and results:**

A retrospective study of patients with NIDCM in AF referred to a single centre for transthoracic echocardiography (TTE) between 2015 and 2019. Peak atrial longitudinal strain (PALS) was measured along with LA emptying fraction and LA filling index (LAFI = E wave/PALS). Cox regression analysis was conducted. A total of 153 patients were included [median age 74 years, left ventricular ejection fraction (LVEF) 35%], and 57 (37.3%) had MACE after a median follow-up of 3.2 years. LAFI was the only independent TTE parameter associated with MACE after adjustment for age, diabetes, LVEF, left ventricular global longitudinal strain (LV-GLS), and LA volume index [adjusted hazard ratio (HR) = 1.02 per point increase, *P* = 0.024], with the best cut-off at ≥15. LAFI ≥15 predicted each of MACE components when separately analysed: MACE HR = 1.95, 95% confidence interval (CI) 1.16–3.30; cardiovascular death HR = 3.68, 95% CI 1.41–9.56, heart failure admission HR = 2.13, 95% CI 1.19–3.80, and ventricular arrhythmia HR = 4.72, 95% CI 1.52–14.67. Higher LAFI was associated with worsening LV-GLS, *E*/*e*′, systolic pulmonary artery (PA) pressure, tricuspid annular plane systolic excursion, and right ventricular to PA coupling.

**Conclusion:**

LA deformation analysis is feasible in patients with NIDCM presenting with AF. LAFI may identify patients at higher risk of MACE and correlates with higher pulmonary pressures and worse right ventricular function, suggesting an elevation of left-sided ventricular pressures in patients with higher LAFI.

## Graphical Abstract

**Graphical Abstract:**
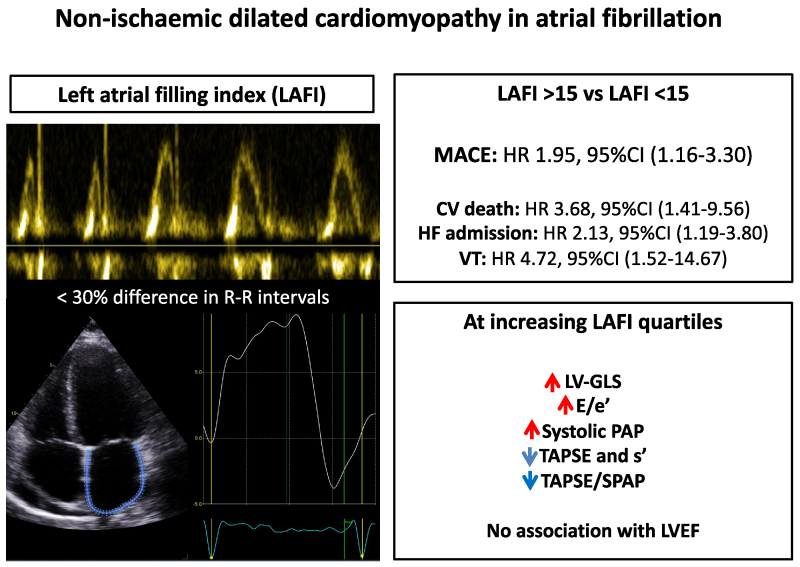

## Introduction

Non-ischaemic dilated cardiomyopathy (NIDCM) is a major cause of heart failure (HF) with reduced left ventricular ejection fraction (LVEF).^1,2^ Atrial fibrillation (AF) is a common comorbidity, encountered in close to 40% of patients with NIDCM,^3^ and it is invariably associated with higher rates of cardiovascular death, admission due to worsening HF symptoms and ventricular arrhythmias.^4–6^

Transthoracic echocardiography (TTE) is cornerstone in the assessment of NIDCM, and several imaging predictors have been described to identify patients at higher risk, such as left ventricular global longitudinal strain (LV-GLS) or left atrial (LA) peak longitudinal strain (PALS) during the reservoir or atrial contraction (PACS) phases.^7–10^ In patients with HF in sinus rhythm, indexes of diastolic dysfunction, such as the *E*/*e*′ ratio, LAVI, PALS, or LA filling index (LAFI), have a better correlation with cardiac events than parameters assessing systolic function.^7–9,11,12^ These indexes, although widely used, have not been properly validated in AF when LVEF is reduced.

Although AF may precipitate and aggravate LA myopathy through electric and anatomic remodelling, in many cases, it represents a late stage after progressive LA scarring and fibrosis with marked diastolic dysfunction, and identifying echocardiographic variables associated with clinical events is challenging in this population. Currently, there is very little data obtained from TTE that can be used to stratify the risk of HF-related events in patients who are in AF, as patients in AF are usually excluded from most observational studies based on beat-to-beat variability.^7–9^ However, some studies including patients in AF with preserved LVEF suggest that these indexes may be useful for prognostic purposes.^13,14^ Therefore, the aim of the current study was to determine whether LA analysis using TTE added prognostic value to the assessment of patients with NIDCM in AF when added to other clinical and echocardiographic variables.

## Methods

### Patient population

We conducted a retrospective cohort study in which we included consecutive patients with NIDCM referred for a TTE between January 2015 and December 2019 to Vall d’Hebron University Hospital, a tertiary referral hospital in Barcelona, Spain. NIDCM was defined as per current cardiomyopathy guidelines as evidence of LV dilatation with global or regional systolic dysfunction with LVEF <50% at the time of the TTE, not explained by coronary artery disease in an invasive coronary angiography.^15,16^ We included patients who were in AF at the time of TTE. We collected baseline characteristics at the time of TTE, as well as comorbidities, medical treatment, and TTE conventional parameters. Patients for whom LA deformation analysis was not available due to poor image quality and those with a medical history of AF but who were in sinus rhythm during TTE were excluded from the analysis. The primary endpoint was time until a major cardiovascular event (MACE), a combination of cardiovascular death, HF admission, or ventricular arrhythmias (sudden cardiac death, resuscitated cardiac arrest, ventricular tachycardia, or appropriate defibrillator shocks). Secondary endpoints were each of the MACE components, as well as non-cardiovascular death. Outcomes were reviewed in parallel by two cardiologists, and a third cardiologist would assign the endpoint in case of discordance. None of the cardiologists collecting outcomes were aware of the values of LA deformation parameters. Follow-up started on the date of the TTE study and ended at the time of death or when last medical contact was available. Follow-up was updated in April 2022 using the electronic health record, and patients were censored at the date of last follow-up if they were still alive. Sudden deaths and those related to HF, acute coronary syndrome, or arrhythmia were considered as cardiovascular deaths. The study was approved by the local investigational review board (PR(AG)379/2022) and follows the Declaration of Helsinki.

### Echocardiographic study

The TTE studies were performed using a Vivid S70, E9, or E95 (GE Healthcare, USA), and images were analysed offline using the Echopac software, version 203 (GE Healthcare). Only patients for whom LA deformation analysis could be completed were included in the study. For strain analysis, 50–70 fps planes were required, and the R wave was set as the starting point, as recommended by clinical practice guidelines.^17^ The atrial myocardium was delimited by tracing a 4 mm region, excluding pulmonary veins. PALS was measured using a dedicated software for the LA using the R wave as the reference (Echopac v203, GE Healthcare), and was defined as the average value of the LA strain measured in the six segments of the four-chamber view.^18^ Maximum and minimum LA volumes were obtained at the end of the ventricular systole and at the end of the ventricular diastole in the four- and two-chamber views using Simpson’s discs method, avoiding views with foreshortening, and both were indexed by body surface area. LA emptying fraction was calculated as the maximum change in LA volume along the cardiac cycle and divided by the maximum LA volume. LAFI was defined as the ratio between transmitral early diastolic filling velocity (E wave, in cm/s) divided by PALS and expressed as a ratio without units.^11^ To measure PALS, we only used a single beat, chosen as the beat with the R–R interval length that was closest to the R–R interval length used for measuring the E wave, accepting a maximum 30% difference between beats. Right ventricular (RV) to pulmonary artery (PA) coupling was defined using the tricuspid annular plane systolic excursion (TAPSE)/systolic PA pressure ratio, and was also measured in beats no more than 30% different in their R–R length. A single operator (E.R.-A.) performed all measurements.

### Statistical analysis

Categorical variables are given as counts and percentages. Given their skewed distribution and significant Shapiro–Wilk test, continuous variables are given as median and interquartile range. Comparisons between categorical variables were performed using the χ^2^ test and Mann–Whitney *U* test. Proportional hazards Cox regression was used to identify variables associated with MACE in univariate analysis. The proportional hazards assumption was ascertained using log–log plots and Schoenfield residuals. A backwards stepwise Cox regression was used to identify the best model for MACE (inclusion criteria *P* < 0.05, exclusion criteria *P* > 0.1), and additional multivariate analyses were performed based on clinically relevant variables. Youden’s index was used to determine the best cut-off for continuous variables. Log-rank test and Kaplan–Meier plots were used to test for differences in event-free survival. Kruskal–Wallis test was used to test for differences among continuous variables by LAFI quartiles. A two-tailed *P*-value of <0.05 was considered significant for all comparisons. All analyses were conducted using Stata 15.0 (StataCorp, US) for Mac.

## Results

**Figure 1:**
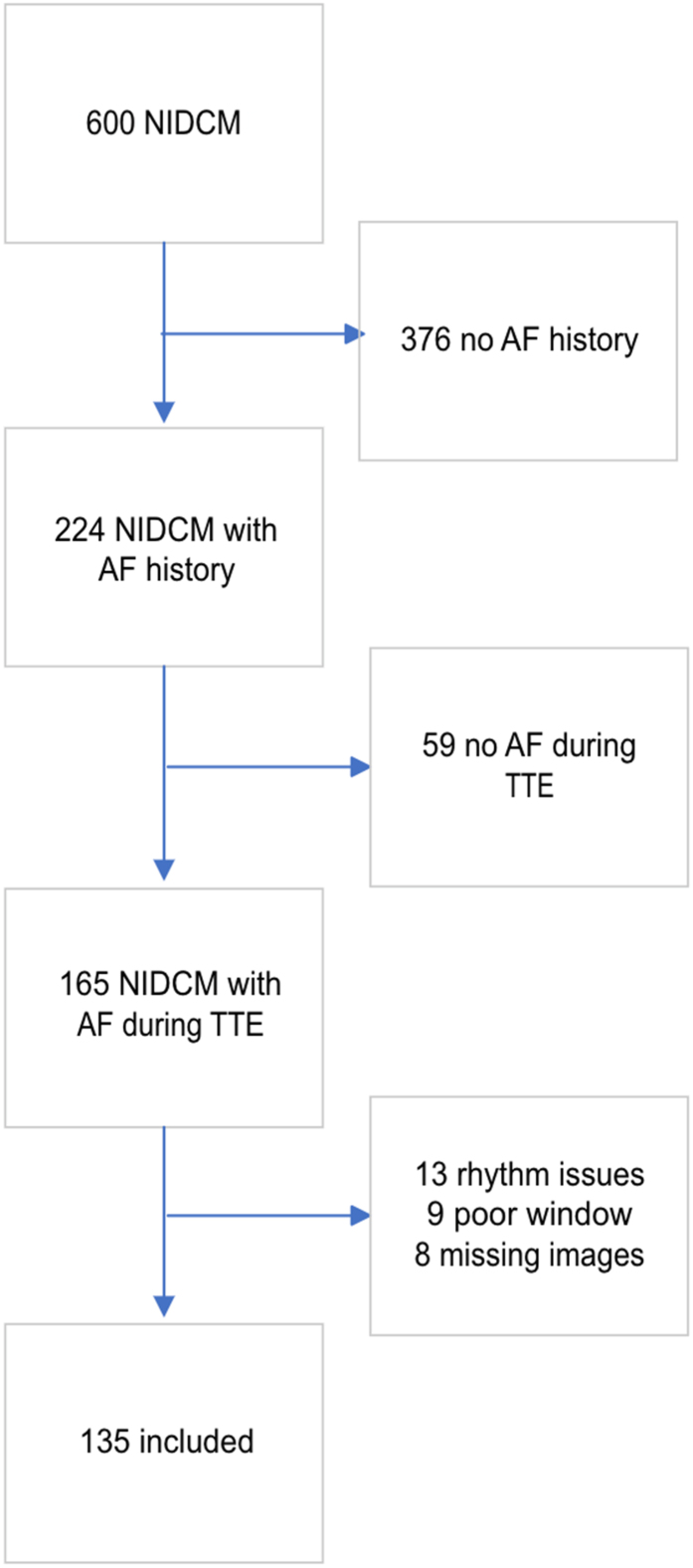
Patient flow chart.

There were 600 patients with NIDCM during the inclusion period, and 224 had a history of AF. However, there were 59 who presented with sinus rhythm during index TTE and were excluded from analysis. Among the 165 remaining patients, complete LA deformation analysis could be completed in 135 patients (82% among those eligible for inclusion, *Figure 1*).

The median age was 74 years, and 48 (31.4%) were female. After a median follow-up of 3.2 years, the primary outcome occurred in 57 (37.3%) patients. There were 21 (13.7%) cardiovascular deaths, 47 (30.7%) patients admitted for HF, and 16 (10.5%) experiencing ventricular arrhythmias. *Table 1* shows baseline characteristics based on the occurrence of the primary endpoint. Patients with MACE were older, with higher symptom burden and on higher doses of diuretic. Diastolic function, using either *e*′ or *E*/*e*′ ratio, was worse in patients with MACE. They also had higher PA pressures and worse LA emptying fraction and higher LAFI, with a non-significant trend towards more impaired PALS.

**Table 1:** Baseline demographics and patient characteristics based on the primary endpoint

	Total (*n* = 153)	No MACE (*n* = 96)	MACE (*n* = 57)	*P*-value
Age (years)	73.7 (64.2–80.3)	71.4 (61.3–78.0)	75.7 (69.9–81.8)	0.005
Female sex	48 (31.4%)	32 (33.3%)	16 (28.1%)	0.50
BMI (kg/m^2^)	27.8 (24.5–31.4)	27.9 (24.4–31.8)	27.2 (25.0–30.1)	0.67
Hypertension	112 (73.2%)	68 (70.8%)	44 (77.2%)	0.39
Dyslipidaemia	71 (47.3%)	40 (42.6%)	31 (55.4%)	0.13
Smoker				0.082
Yes	23 (15.1%)	19 (20.0%)	4 (7.0%)	
No	72 (47.4%)	44 (46.3%)	28 (49.1%)	
Previous smoker	57 (37.5%)	32 (33.7%)	25 (43.9%)	
Diabetes mellitus	43 (28.5%)	23 (24.5%)	20 (35.1%)	0.16
Stroke	20 (13.2%)	9 (9.4%)	11 (19.6%)	0.071
HFH previous year	72 (48.6%)	48 (51.1%)	24 (44.4%)	0.44
VT previous year	4 (2.7%)	1 (1.1%)	3 (5.5%)	0.12
NYHA at baseline				0.003
I	30 (22.1%)	26 (28.9%)	4 (8.7%)	
II	92 (67.6%)	59 (65.6%)	33 (71.7%)	
III	14 (10.3%)	5 (5.6%)	9 (19.6%)	
Heart rate (bpm)	80.0 (70.0–100.0)	85.0 (70.0–110.0)	80.0 (70.0–90.0)	0.079
Systolic BP (mmHg)	121.0 (111.0–133.0)	123.0 (111.0–134.0)	120.0 (110.0–130.0)	0.31
Diastolic BP (mmHg)	72.0 (64.0–80.0)	74.5 (67.0–80.0)	70.0 (60.0–79.0)	0.054
Furosemide	87 (57.2%)	48 (50.5%)	39 (68.4%)	0.031
ACEI, ARB or ARNI	115 (75.2%)	77 (80.2%)	38 (66.7%)	0.061
Beta-blocker	135 (88.2%)	90 (93.8%)	45 (79.0%)	0.006
MRA	85 (55.6%)	57 (59.4%)	28 (49.1%)	0.22
LVEF (%)	35.0 (31.0–41.0)	35.0 (29.5–40.0)	38.0 (32.0–45.0)	0.052
LVEDD (mm)	55.0 (49.0–60.0)	56.0 (49.0–60.0)	53.0 (46.0–62.0)	0.60
LVEDVi (mL/m^2^)	62.2 (51.2–80.4)	62.8 (51.3–77.0)	60.5 (50.8–94.3)	0.73
LVESVi (mL/m^2^)	39.3 (31.7–54.4)	40.6 (32.0–50.4)	38.7 (29.3–59.9)	0.63
LV-GLS (%)	−10.0 (−12.4 to 7.6)	−10.0 (−12.4 to 7.5)	−10.1 (−12.4 to 8.1)	0.91
TAPSE (mm)	16.0 (14.0–17.0)	16.0 (14.0–18.0)	15.0 (13.5–17.0)	0.17
E wave (m/s)	1.0 (0.8–1.2)	0.9 (0.8–1.1)	1.0 (0.8–1.3)	0.067
*e*′ (cm/s)	7.0 (5.5–9.5)	8.0 (6.0–9.5)	6.0 (4.5–7.0)	0.018
*E*/*e*′ ratio	12.6 (9.1–19.0)	10.6 (8.9–18.2)	17.7 (11.9–23.1)	0.031
LAVI (mL/m^2^)	48.6 (37.1–66.4)	45.8 (36.7–63.3)	53.7 (41.4–68.8)	0.10
PALS (%)	8.0 (5.0–10.0)	8.0 (6.0–10.0)	7.0 (5.0–9.0)	0.053
LA EF (%)	23.0 (17.0–30.0)	24.0 (19.0–31.0)	21.0 (15.0–27.0)	0.017
LAFI	12.8 (9.1–20.2)	11.7 (8.8–17.7)	16.4 (9.3–31.7)	0.015
Systolic PAP (mmHg)	40.0 (31.0–50.0)	35.5 (29.0–44.0)	46.0 (35.5–56.0)	<0.001
TAPSE/systolic PAP	0.4 (0.3–0.6)	0.4 (0.3–0.6)	0.3 (0.2–0.4)	<0.001

MACE, major cardiovascular adverse events; BMI, body mass index; HFH, heart failure hospitalization; VT, ventricular tachycardia; NYHA, New York Heart Association; BP, blood pressure; ACEI, angiotensin-converting enzyme inhibitor; ARB, angiotensin receptor blocker; ARNI, angiotensin receptor/neprilysin inhibitor; MRA, mineralocorticoid receptor antagonist; LVEF, left ventricular ejection fraction; LVEDD, left ventricular end-diastolic diameter; LVEDVi, left ventricular end-diastolic volume index; LVESVi, left ventricular end-systolic volume index; LV-GLS, left ventricular global longitudinal strain; TAPSE, tricuspid annular plane systolic excursion; PALS, peak atrial longitudinal strain; LAFI, left atrial filling index; EF, emptying fraction; LAVI, left atrial volume index; PAP, pulmonary artery pressure.

*Table 2* shows the results of the proportional hazards Cox regression. Variables with a *P*-value <0.1 in univariate analysis were included in a backwards stepwise regression, resulting in a multivariate model that only included age and LAFI as independently associated with MACE, with a C-statistic = 0.65 for the endpoint. Among TTE parameters, only LAFI remained in the model. To identify whether LAFI identified a different remodelling phenomenon than LA enlargement, a two-term interaction between LAFI and LAVI was tested with non-significant results (*P* for interaction = 0.31). In this analysis, LAFI remained associated with MACE [hazard ratio (HR) = 1.03 per point increase, 95% confidence interval (CI) 1.01–1.05], whereas LAVI did not (HR = 1.00 per mL/m^2^ increase, 95% CI 0.99–1.01). The association between LAFI and MACE also remained after adjustment for clinically relevant covariates (age, diabetes, LVEF, LV-GLS, and LAVI), with an adjusted HR = 1.02 (95% CI 1.00–1.04, *P* = 0.024).

**Table 2:** Univariate and multivariate analysis of predictors

	Univariate analysis	Multivariate model
Age (years)	1.05 (1.02–1.08)	1.05 (1.01–1.08)
Female sex	0.81 (0.45–1.45)	
Hypertension	1.29 (0.70–2.40)	
Dyslipidaemia	1.61 (0.95–2.73)	
Diabetes mellitus	1.73 (1.00–2.99)	
HFH in previous year	0.84 (0.49–1.44)	
VT previous year	3.14 (0.97–10.14)	
Heart rate (bpm)	0.99 (0.98–1.00)	
Systolic BP (mmHg)	0.99 (0.97–1.01)	
Diastolic BP (mmHg)	0.98 (0.96–1.00)	
LVEF (%)	1.03 (1.00–1.06)	
LV-GLS (%)	1.02 (0.93–1.12)	
TAPSE (mm)	0.95 (0.87–1.03)	
*E*	1.59 (0.91–2.78)	
*e*′	0.79 (0.64–0.98)	
*E*/*e*′ ratio	1.04 (1.01–1.08)	
LAVI	1.00 (1.00–1.01)	
PALS (%)	0.93 (0.85–1.01)	
LA EF (%)	0.97 (0.94–1.00)	
LAFI	1.02 (1.01–1.04)	1.02 (1.01–1.04)
Systolic PAP (mmHg)	1.04 (1.02–1.06)	

HFH, heart failure hospitalization; VT, ventricular tachycardia; BP, blood pressure; LVEF, left ventricular ejection fraction; LV-GLS, left ventricular global longitudinal strain; TAPSE, tricuspid annular plane systolic excursion; PALS, peak atrial longitudinal strain; LAFI, left atrial filling index; EF, emptying fraction; LAVI, left atrial volume index; PAP, pulmonary artery pressure.

**Figure 2:**
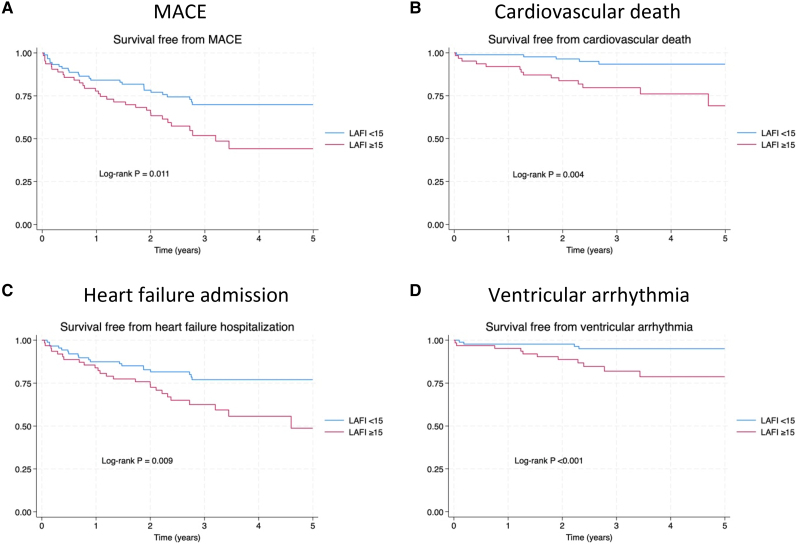
Survival free from each endpoint based on LA filling index. Log-rank *P*-values: MACE = 0.011. Ventricular arrhythmia <0.001. HF admission = 0.009. CV death = 0.003. (*A*) MACE. (*B*) Cardiovascular death. (*C*) Heart failure admission. (*D*) Ventricular arrhythmia.

The best cut-off for LAFI to identify patients at the highest risk of MACE was ≥15. Patients were divided best on this cut-off as LAFI ≥15 and LAFI <15. *Table 3* shows that patients with LAFI ≥15 more frequently had a history of diabetes and were more symptomatic. LV-GLS, TAPSE, *E*/*e*′ ratio, and systolic PA pressure were worse in the LAFI ≥15 group, but LVEF was similar between groups. Event rates for MACE were higher in patients with LAFI ≥15 (20.9 vs. 9.7 MACE per 100 person-years, HR = 1.95, 95% CI 1.16–3.30; *Table 4*). When MACE components were separately analysed, LAFI ≥15 remained associated with each of them: cardiovascular death HR = 3.68, 95% CI 1.41–9.56 (log-rank *P* = 0.003), HF admission HR = 2.13, 95% CI 1.19–3.80 (log-rank *P* = 0.009), and ventricular arrhythmia HR = 4.72, 95% CI 1.52–14.67 (log-rank *P* < 0.001; *Figure 2*). Non-cardiovascular death rates were similar between groups (log-rank *P* = 0.22).

**Table 3:** Baseline characteristics based on high or low LA filling index

	Total (*n* = 153)	LAFI <15 (*n* = 89)	LAFI ≥15 (*n* = 64)	*P*-value
Age (years)	73.7 (64.2–80.3)	73.6 (61.4–79.0)	75.1 (67.1–80.7)	0.11
Female sex	48 (31.4%)	23 (25.8%)	25 (39.1%)	0.082
BMI (kg/m^2^)	27.8 (24.5–31.4)	28.2 (24.5–31.6)	27.7 (24.5–30.3)	0.62
Hypertension	112 (73.2%)	60 (67.4%)	52 (81.2%)	0.057
Dyslipidaemia	71 (47.3%)	40 (46.0%)	31 (49.2%)	0.70
Smoker				0.24
Yes	23 (15.1%)	16 (18.2%)	7 (10.9%)	
No	72 (47.4%)	37 (42.0%)	35 (54.7%)	
Previous smoker	57 (37.5%)	35 (39.8%)	22 (34.4%)	
Diabetes mellitus	43 (28.5%)	18 (20.2%)	25 (40.3%)	0.007
Stroke	20 (13.2%)	9 (10.1%)	11 (17.5%)	0.19
HFH previous year	72 (48.6%)	38 (44.7%)	34 (54.0%)	0.26
VT previous year	4 (2.7%)	2 (2.4%)	2 (3.2%)	0.77
NYHA at baseline				0.017
I	30 (22.1%)	24 (30.4%)	6 (10.5%)	
II	92 (67.6%)	49 (62.0%)	43 (75.4%)	
III	14 (10.3%)	6 (7.6%)	8 (14.0%)	
Heart rate (bpm)	80.0 (70.0–100.0)	80.0 (70.0–100.0)	80.0 (70.0–107.5)	0.86
Systolic BP (mmHg)	121.0 (111.0–133.0)	120.0 (110.0–131.0)	123.0 (115.0–135.0)	0.21
Diastolic BP (mmHg)	72.0 (64.0–80.0)	72.5 (65.0–80.0)	72.0 (60.0–80.0)	0.92
Furosemide	87 (57.2%)	46 (51.7%)	41 (65.1%)	0.10
ACEI, ARB, or ARNI	115 (75.2%)	70 (78.7%)	45 (70.3%)	0.24
Beta-blocker	135 (88.2%)	84 (94.4%)	51 (79.7%)	0.005
MRA	85 (55.6%)	48 (53.9%)	37 (57.8%)	0.63
LVEF (%)	35.0 (31.0–41.0)	37.0 (31.0–41.0)	34.5 (31.0–41.0)	0.33
LVEDD (mm)	55.0 (49.0–60.0)	55.5 (50.5–60.0)	55.0 (47.0–61.0)	0.56
LVEDVi (mL/m^2^)	62.2 (51.2–80.4)	59.9 (48.8–75.1)	66.3 (53.7–94.2)	0.034
LVESVi (mL/m^2^)	39.3 (31.7–54.4)	38.7 (29.7–49.8)	39.5 (34.0–62.1)	0.080
LV-GLS (%)	−10.0 (−12.4 to 7.6)	−10.9 (−12.9 to 7.9)	−9.9 (−11.3 to 7.0)	0.040
TAPSE (mm)	16.0 (14.0–17.0)	16.0 (14.0–18.0)	15.0 (14.0–17.0)	0.012
E wave	1.0 (0.8–1.2)	0.9 (0.8–1.0)	1.2 (0.9–1.6)	<0.001
*e*′	7.0 (5.5–9.5)	8.0 (6.0–9.5)	5.8 (5.0–7.0)	0.013
*E*/*e*′ ratio	12.6 (9.1–19.0)	10.5 (8.9–13.3)	20.9 (17.6–23.2)	<0.001
LAVI	48.6 (37.1–66.4)	42.2 (32.8–53.2)	60.7 (48.2–83.3)	<0.001
PALS (%)	8.0 (5.0–10.0)	9.0 (8.0–11.0)	5.0 (4.0–6.0)	<0.001
LA EF (%)	23.0 (17.0–30.0)	27.0 (22.0–34.0)	17.0 (14.0–22.0)	<0.001
LAFI	12.8 (9.1–20.2)	9.3 (7.5–12.0)	22.6 (17.7–34.4)	<0.001
Systolic PAP (mmHg)	40.0 (31.0–50.0)	36.5 (28.0–46.0)	44.0 (35.0–55.5)	0.001
TAPSE/systolic PAP	0.4 (0.3–0.6)	0.4 (0.3–0.6)	0.3 (0.2–0.4)	<0.001

MACE, major cardiovascular adverse events; BMI, body mass index; HFH, heart failure hospitalization; VT, ventricular tachycardia; NYHA, New York Heart Association; BP, blood pressure; ACEI, angiotensin-converting enzyme inhibitor; ARB, angiotensin receptor blocker; ARNI, angiotensin receptor/neprilysin inhibitor; MRA, mineralocorticoid receptor antagonist; LVEF, left ventricular ejection fraction; LVEDD, left ventricular end-diastolic diameter; LVEDVi, left ventricular end-diastolic volume index; LVESVi, left ventricular end-systolic volume index; LV-GLS, left ventricular global longitudinal strain; TAPSE, tricuspid annular plane systolic excursion; PALS, peak atrial longitudinal strain; LA, left atrial filling index; EF, emptying fraction; LAVI, left atrial volume index; PAP, pulmonary artery pressure.

**Table 4:** Event rate for the primary endpoint and each of its components based on LAFI

	LAFI	Event rate (per 100 person-years)	Hazard ratio (95% CI)
MACE	≥15	20.9	1.95 (1.16–3.30)
	<15	9.7	
Cardiovascular death	≥15	7.3	3.68 (1.41–9.56)
	<15	2.0	
Non-cardiovascular death	≥15	5.2	1.73 (0.72–4.16)
	<15	3.3	
HF admission	≥15	16.3	2.13 (1.19–3.80)
	<15	7.2	
Ventricular arrhythmia	≥15	6.0	4.72 (1.52–14.67)
	<15	1.2	

MACE, major cardiovascular adverse events; HF, heart failure; LAFI, left atrial filling index; CI, confidence interval.

**Figure 3:**
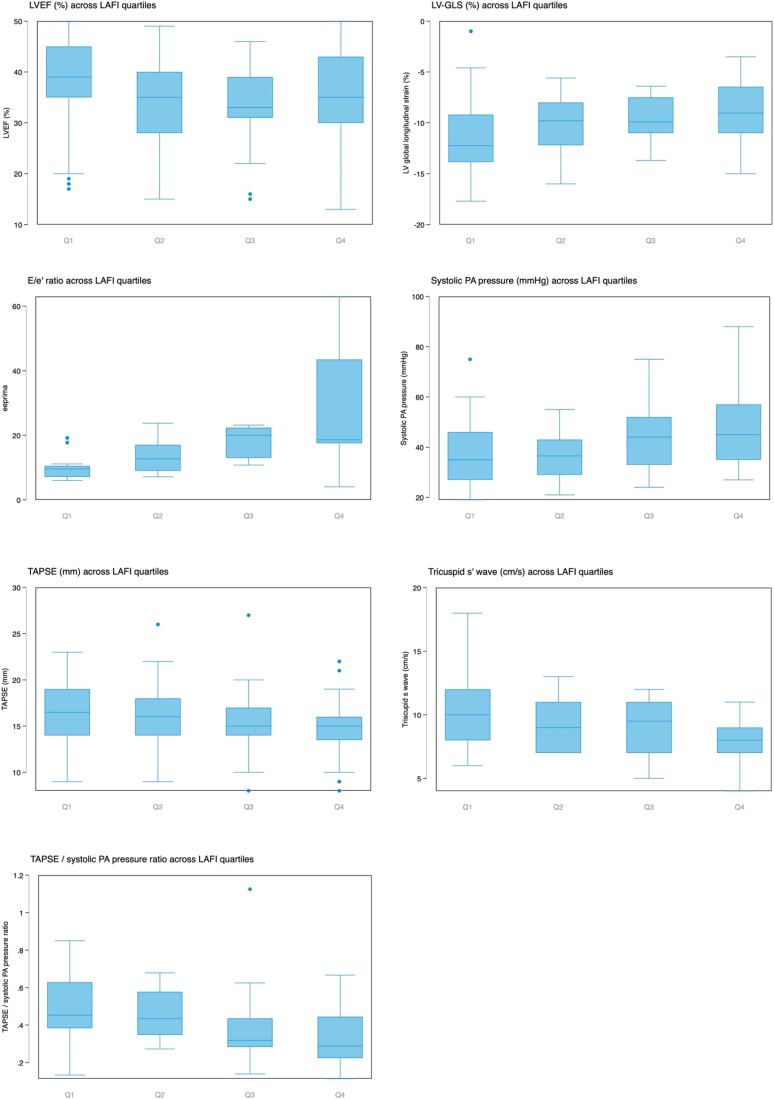
Differences in echocardiographic parameters across LA filling index quartiles. Q1: quartile 1, LAFI 0–9; Q2: quartile 2, LAFI 9–13; Q3: quartile 3, LAFI 13–20; Q4: quartile 4, LAFI >20. *P* for Q1 vs. Q4 for LVEF = 0.18; *P* for LV-GLS = 0.002; *P* for *E*/*e*′ = 0.016; *P* for systolic PAP = 0.008; *P* for TAPSE = 0.006; *P* for tricuspid *s*′ < 0.001; *P* for TAPSE/systolic PAP < 0.001.

*Figure 3* shows the relationship between TTE parameters and LAFI, when divided in quartiles. As LAFI increased, there was a significant worsening in LV-GLS, *E*/*e*′ ratio, and progressive increase in systolic PA pressures, with deleterious effects on RV function, as reflected by decreasing TAPSE and tricuspid *s*′ wave, as well as worsening RV-PA coupling (*P*-value for the difference between groups <0.05 for all comparisons, using Kruskal–Wallis test). LAFI had no relation with LVEF (*Figure 3*).

## Discussion

This study demonstrates that LAFI may identify patients with NIDCM and AF who are at higher risk of cardiovascular death, HF hospitalization, or ventricular arrhythmia, and it correlates with higher PA pressures and worse RV function. This suggests that LA deformation could be incorporated into the routine assessment of patients with AF and NIDCM.

LAFI has a linear association with well-stablished risk factors among patients in sinus rhythm, such as LV-GLS and *E*/*e*′ ratio, but was completely independent from LVEF. Although we do not provide invasive measurements, LAFI was also associated with increased systolic PA pressures, poorer RV function, and a more impaired RV-PA coupling using the TAPSE/systolic PA pressure ratio. We believe that these data are highly suggestive that LAFI probably is a non-invasive correlate of LV filling pressures. At least in patients in sinus rhythm with preserved LVEF, LAFI had very high accuracy to predict elevation of LV filling pressures.^19^

AF sometimes represents a final stage of LA cardiomyopathy, being both a contributor by inducing LA electrical and anatomical remodelling and, at the same time, a consequence of LA scarring and fibrosis, which may in turn favour blood stasis and thrombus formation within the LA that may portend higher risk of stroke. This combined phenomena lead to higher LA stiffness and elevated LA pressures,^20,21^ which may be reflected in abnormal deformation mechanics and invasively correlated with large V waves in the capillary wedge tracing in the absence of mitral regurgitation and has been associated with an elevated risk of HF admission.^22^ Since there is a larger LA preload, E wave will be higher.^23^ Even if the LA is in AF, the preload will generate a pressure that will distend the non-contractile LA to a certain extent, determined by LA wall stiffness and compliance, that is captured by PALS,^24^ which may also be affected by LV-GLS through the mitral annulus displacement.^25^

This relationship between LA preload and LA distension as a response a certain preload may not vary from beat to beat with AF irregular heart rhythm: a faster heartbeat will have a lower preload (measured as a lower E wave), but PALS would also decrease due to a smaller LA wall distension, whereas a slower heartbeat would allow the LA to fill with a larger preload (higher E wave) that would passively distend the LA to a larger extent (higher PALS), therefore resulting in a similar LAFI in both R–R intervals. It is not possible with the current technology to simultaneously measure PALS and E wave using the same heartbeat, but we used the closest R–R intervals available to measure LAFI.

LAFI is not a novel index, and its association with adverse cardiovascular events has been previously reported in patients in sinus rhythm and among those with AF, with similar association with MACE.^11,12,26,27^ However, the only study reporting LAFI in AF included only a minority of patients with reduced LVEF, and the cut-off the authors found to be associated with MACE was lower (LAFI > 6) that ours, likely due to the higher LVEF with larger apical displacement of the mitral annulus during the ventricular systole, resulting in higher PALS and, consequently, lower LAFI.^27^ LAFI in individuals in sinus rhythm with preserved PALS is expectedly higher due to even greater PALS, and a lower cut-off of >3.3 has been suggested to identify patients at risk of MACE.^11^ This variability identifies a gap in knowledge, as it seems imperative to define reference values for PALS and LAFI a range of LVEF before the widespread adoption of these indexes and cut-offs in AF.

Patients with long-standing AF display larger LAVI. LAVI, then, may not reflect filling pressures in patients who are in AF as accurately as it does in those who remain in sinus rhythm, and it performs even worse in the setting of reduced LVEF.^28^ LAVI is also a major determinant of PALS^28^ and, as such, may have mediated the association between LAFI and MACE. To explore the association between LAVI and LAFI, we tested a two-term interaction between both that was not significant, and only LAFI, but not LAVI, remained with MACE in adjusted analyses. Thus, LAFI could be equally useful to identify patients at higher risk of MACE among those with long-standing AF and atrial dilatation.

LAFI is easy to obtain from a four-chamber view from the E wave and PALS, and it is not time-consuming. It can be retrospectively measured from stored images, as most patients would have these images already acquired, and it is a parameter that is not based on any assumptions, and only requires to measure the closer R–R intervals for both E wave and PALS. It is based on speckle tracking and not on Doppler and is, therefore, independent from the angle, which makes it highly reproducible.^14^ Unlike systolic PA pressure, it does not require tricuspid regurgitation and can be measured any time that a four-chamber view with enough image quality is obtained, using a dedicated software. Thus, in the light of these results, we advocate for the inclusion of LA deformation, and LAFI in particular, to the routinary TTE assessment of NIDCM in AF, even if there is a lack of reference values in otherwise healthy patients. In the setting of an elevated LAFI, it may seem reasonable to intensify disease-modifying medication, if feasible, or performing a closer follow-up given the higher risk of events.

### Limitations

This is a retrospective, single-centre study with a limited sample size and, as such, can only be seen as hypothesis generating. We do not have invasive pressure measurements obtained from right heart catheterizations, so that we cannot ascertain the association between LAFI and elevated filling pressures. However, the association between LAFI and the rest of TTE parameters and MACE during follow-up are very suggestive of this correlation. Although AF presents significant beat-to-beat variation in R–R intervals, the fact that a single operator (E.R.-A.) performed all the analysis using similar R–R intervals for both E wave and PALS ensures the consistency of the reported results.

## Conclusion

LAFI could be useful in patients with NIDCM who are in AF during TTE acquisition to identify those at higher risk of MACE during follow-up after accounting for LAVI, with an optimal cut-off at ≥15. Higher LAFI correlates with worse LV-GLS, higher systolic PA pressures, worse RV function and more impaired RV-PA coupling, suggesting an association with higher LV filling pressures.

## Data Availability

Data will be available upon reasonable request.

## Lead author biography

Eduard Ródenas-Alesina is a Cardiologist at Vall d‘Hebron University Hospital, Barcelona, where he works in the in-patient ward and as part of the Heart Failure Unit. He trained in Toronto General Hospital as a fellow in advanced Heart Failure and Transplantation.
